# Role of TSP-1 as prognostic marker in various cancers: a systematic review and meta-analysis

**DOI:** 10.1186/s12881-020-01073-3

**Published:** 2020-06-29

**Authors:** Shengjie Sun, Huiyu Dong, Tao Yan, Junchen Li, Bianjiang Liu, Pengfei Shao, Jie Li, Chao Liang

**Affiliations:** Department of Urology, The First Affiliated Hospital of Nanjing Medical University, Nanjing, China

**Keywords:** TSP-1, Malignant neoplasm, Prognosis, Overall survival, Meta-analysis

## Abstract

**Background:**

Published studies present conflicting data regarding the impact of Thrombospondin-1 (TSP-1) expression on prognosis of various cancers. We performed this meta-analysis to illustrate the preliminary predictive value of TSP-1.

**Methods:**

Twenty-four studies with a total of 2379 patients were included. A comprehensive literature search was performed by using PubMed, Cochrane Library, Web of Science, Embase, and hand searches were also conducted of relevant bibliographies. Pooled hazard ratios (HRs) with 95% confidence intervals (CIs) for patient survival and disease recurrence were initially identified to explore relationships between TSP-1 expression and patient prognosis.

**Results:**

A total of 24 eligible studies were included in this meta-analysis. Our results showed that high level of TSP-1 was correlated significantly with poor overall survival (OS) (HR = 1.40, 95% CI: 1.17 ~ 1.68; P<0.001). However, high TSP-1 expression predicted no significant impact on progression-free survival (PFS)/ metastasis-free survival (MFS) (HR = 1.35, 95%CI: 0.87–2.10; *P* = 0.176) and disease-free survival (DFS)/ recurrence-free survival (RFS) (HR = 1.40, 95%CI: 0.77–2.53; *P* = 0.271). In addition, we performed subgroup analyses which showed that high TSP-1 expression predicted poor prognosis in breast cancer and gynecological cancer. Additionally, the relatively small number of studies on PFS/MFS and DFS/RFS is a limitation. The data extracted through Kaplan-Meier curves may not be accurate. Moreover, only English articles were included in this article, which may lead to deviations in the results.

**Conclusions:**

Our findings indicated high TSP-1 expression may act as a promising biomarker of poor prognosis in cancers, especially in breast cancer and gynecological cancer.

## Background

Thrombospondin-1 (TSP-1) is one of the thrombospondin gene family, which be composed of five matricellular proteins includes TSP-1, − 2, − 3, − 4, and − 5 [1, 2]. Thrombospondin gene family plays a role in extensive physiological and pathological processes, including development, angiogenesis, inflammation and neoplasia [3]. This TSP family interact with a variety of membrane proteins on the cell surface, such as proteoglycans, integrins, CD36 and CD47 [4, 5]. Thus, during tissue development and remodeling, TSP family control cellular phenotype and extracellular matrix structure [3].

TSP-1 is a multifunctional matrix glycoprotein which is synthesized and secreted by platelets, endothelial cells and smooth muscle cells. Under the transmission microscope, TSP-1 is composed of three identical peptide chains, each showing a spherical amino at one end and a spherical carboxyl at the other end, connected by a slender rod-like arm in the middle [6]. Because of multiple functional domains, TSP-1 can mediate the interaction between cell and cell, cell and extracellular matrix. Therefore, TSP-1 is a kind of glycoprotein with a wide range of biological effects, such as activating transforming growth factor-β, inhibiting angiogenesis, anti-tumor activity, participating in tissue repair and so on [7, 8]. TSP-1 was originally found in platelets, but now it has been shown to play an important role in carcinogenesis [9, 10]. Besides its direct role in regulating the behavior of tumor cells, TSP-1 also shows function in tumor vessels [11]. Taken together, TSP-1 can regulate the growth, adhesion and migration of tumor cells [12].

The function of TSP 1 remains disputable in angiogenesis and tumor progression. In some cancers, TSP 1 has been deemed to be an inhibitor of both processes [13–15], while in others, it has been considered a stimulator [16–18]. Some research concluded that the actual function of TSP 1 was organ specific [19].

To address this issue, we performed meta-analysis to comprehensively assess the overall risk of TSP-1 for survival in patients with various types of cancers. Furthermore, we attempt to evaluate the value of TSP-1 as a prognostic marker in the aspect of clinical features and statistics.

## Methods

### Search strategy

Original studies aimed to analyze the predictive value of TSP-1 in multiple human malignant neoplasms. PubMed, Web of Science, Cochrane Library and Embase were searched up from inception to December 14, 2019 using the following key words: “Thrombospondin 1”, “Neoplasm”, “prognosis”, “survival”, “recurrence”, “death”, “incidence”, “mortality”. The search strategy used a combination of Medical Subject Headings and thesaurus terms.

### Inclusion criteria and exclusion criteria

We considered articles were considered qualified when they met the following criteria: (1) patients diagnosed with cancers by using pathological methods (2) studies focusing on the relationship between TSP-1 expression and prognosis, (3) survival outcomes with 95% confidence intervals (CIs) and hazard ratios (HRs) that could be extracted directly or indirectly. Excluding criteria were as follows: (1) Not a human study; (2) Not original articles; (3) Unrelated to TSP-1; (4) No clinical parameters; (5) Unrelated to malignant tumor; (6) Not related to prognosis or survival; (7) Insufficient survival data; (8) Overlapping data.

### Quality assessment

Quality assessment of the included studies was conducted independently by three researchists (SS, HD and TY) to rule out any discrepancy. Studies for inclusion include the following criteria: (1) the study country and population; (2) definition of study design (3) the samples and pathology information; (4) defiition of measurement of TSP-1; (5) the clinical outcomes and follow-up duration.

### Data extraction

All relevant studies were identified by two independent reviewers (TY and HD), and any disagreements were reassessed by a third reviewer (SS). The data elements include the following information: (1) publication year and first author’s name; (2) nationality, dominant ethnicity, number of patients, sample type, and main type of pathology; (3) Assay method, follow-up time; (4) TSP-1 expression levels and cut-off values; (5) HRs related to elevated TSP-1 expression for overall survival (OS), recurrence-free survival (RFS), and disease-free survival (DFS). Those indirectly reported HRs and 95% CIs were calculated using graphical survival plots.

### Statistical methods

A random or fixed model was selected according the heterogeneity which was measured by the Q statistics and Higgins I-squared statistic (I^2^). If *P* < 0.10 or I^2^ > 50%, a random-effects model was applied and subgroup ana lyses was carried out to fine the source of heterogeneity; otherwise, a fixed-effects model was adopted [20]. Publication bias was assessed by Egger’s test and Begg’s funnel plot [21]. To examine the stability and dependability of the overall outcomes, sensitivity analyses were performed by excluding one single study one by one and recalculating their HRs. All *P*-values were calculated using a two-sided test. We consider *P* < 0.05 as statistically significant. All statistical analyses were conducted by using Excel software 2016 and Stata version 12.0.

## Results

### Study selection

**Fig. 1:**
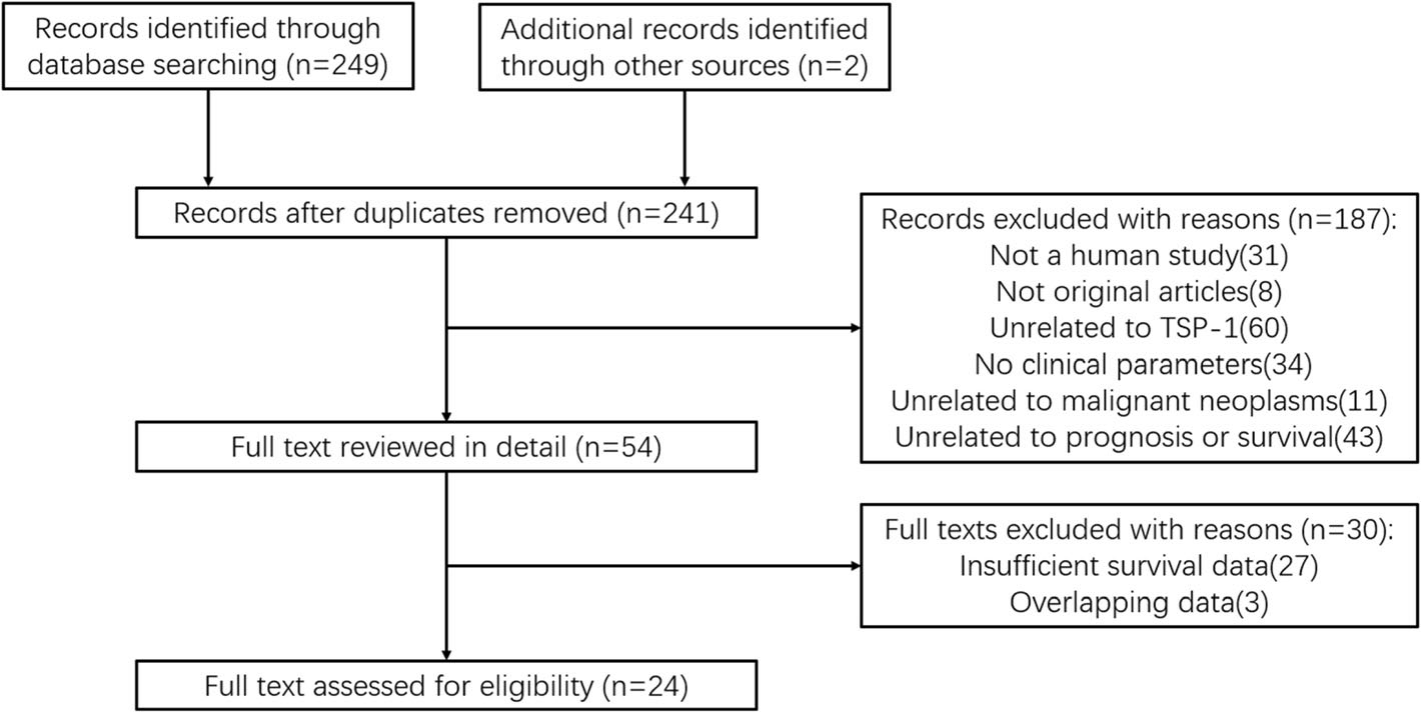
Selection process of studies for meta-analysis

The flow chart of study selection process was depicted in Fig. 1. A total of 251 studies were identified from online databases, including PubMed, Cochrane Library, Web of Science and Embase. Based on screening of titles and abstracts, 54 studies were selected for further investigation according to following criteria: repetitive articles, not a human study, not original articles, no clinical parameters, unrelated to TSP-1, unrelated to malignant neoplasms and unrelated to prognosis or survival. Of these 54 studies, 30 were excluded due to insufficient survival data and overlapping data. Ultimately, 24 articles were included for further analysis.

### Study characteristics

The major characteristics of the eligible data were summarized in Table 1. We collected basic data on 24 articles published between 2000 and 2019. The meta-analysis included 2379 participants from different regions of the United States, France, Japan, China, India, Greece, the United Kingdom and Norway, including tumors such as breast cancer, liver cancer, ovarian cancer, esophagus cancer, lung cancer, gastric cancer, colon cancer, skin cancer, cervical cancer, oral cancer and bladder cancer.

**Table. 1:** Main characteristics of studies included in the meta-analysis

First author, Publication year	Case nationality	Male (%)	Domi nant ethnicity	Main pathological type	Disease type	Detec ted sample	Outco me measures	Sourc e of HR	Maxi mum months of follow-up	Assay method
Nakamura et al., 2019	Japan	0.79	Asian	Urothelial carcinoma	Bladder cancer	Tissue	PFS	Reported	95	IHC
Tzeng et al., 2016	China	N/A	Asian	Squamous cell carcinoma	Esophagus cancer	Tissue	PFS	SC	228	IHC
Rouanne et al., 2016	France	0.67	Caucasian	Adenocarcinoma	Lung cancer	Serum	OS/DFS	Reported	34	ELISA
Teraoku et al., 2016	Japan	0.35	Asian	Adenocarcinoma	Colon cancer	Tissue	OS/DFS	SC	168	IHC
Campone et al., 2015	France	N/A	Caucasian	Adenocarcinoma	Breast cancer	Tissue	OS	SC	279	IHC
Eto et al., 2015	Japan	0.8	Asian	Adenocarcinoma	Gastric cancer	Tissue	OS	SC	60	IHC
Yao et al., 2014	China	0.68	Asian	Non-Small Cell Lung Cancer	Lung cancer	Tissue	OS/DFS	Reported	60	ELISA
Sharma et al., 2013	India	92.5	Asian	Hepatocellular Carcinoma	Liver cancer	Tissue	OS	Reported	33	PCR
Pectasides et al., 2012	Greece	0	Caucasian	Adenocarcinoma	Breast cancer	Tissue	OS/PFS	Reported	45	PCR
Nakao et al., 2011	Japan	0.8	Asian	Adenocarcinoma	Gastric cancer	Tissue	OS	SC	50	IHC
Zhou et al., 2009	China	0.73	Asian	Squamous cell carcinoma	esophagus cancer	Tissue	OS	Reported	50	IHC
Randall et al., 2009	USA	N/A	Caucasian	Squamous cell carcinoma	Cervical cancer	Tissue	OS/PFS	Reported	184.8	IHC
Yang et al., 2009	China	0.51	Asian	mucoepidermoid carcinoma	Oral cancer	Tissue	DFS	SC	60	IHC
Secord et al., 2007	USA	0	Caucasian	Epithelial cancer	Ovarian cancer	Tissue	OS/PFS	Reported	110	Immunoblot analysis
Wada et al., 2006	Japan	0.75	Asian	Hepatocellular Carcinoma	Liver cancer	Tissue	DFS	SC	60	IHC
Sutton et al., 2005	UK	0.67	Caucasian	Adenocarcinoma	Colon cancer	Tissue	OS	Reported	60	Dextran polymer conjugate wwo-step visualization system
Fontana et al., 2005	France	N/A	Caucasian	Adenocarcinoma	Breast cancer	Tissue	PFS	SC	160	IHC
Poon et al., 2004	China	0.82	Asian	Hepatocellular Carcinoma	Liver cancer	Tissue	OS	SC	38	IHC
Aishima et al., 2002	Japan	N/A	Asian	Intrahepatic Cholangiocarcinoma	Liver cancer	Tissue	OS	SC	120	IHC
Maeda et al., 2001	Japan	0.63	Asian	Adenocarcinoma	Colon cancer	Tissue	DFS	Reported	60	IHC
Straume et al., 2001	Norway	N/A	Caucasian	Melanomas	Skin cancer	Tissue	OS	SC	210	IHC
Kodama et al., 2001	Japan	N/A	Asian	Squamous cell carcinoma	Cervical cancer	Tissue	DFS	Reported	59	PCR
You et al., 2000	China	0.57	Asian	Squamous cell carcinoma	Lung cancer	Tissue	OS	SC	120	IHC
Yao et al., 2000	Japan	N/A	Asian	Squamous cell carcinoma	Oral cancer	Tissue	OS	SC	60	IHC

OS, overall survival; DFS, disease-free survival; PFS, progression-free survival; SC: survival curve; IHC, Immunohistochemistry; PCR, polymerase chain reaction; ELISA, enzyme linked immunosorbent assay; N/A, not available

The expression of TSP-1 was measured by Immunohistochemical staining (IHC) in the most of studies. Besides, Quantitative real-time polymerase chain reaction (qRT-PCR) assay and enzyme linked immunosorbent assay (ELISA) was applied to detect TSP-1 in 3 and 2 studies, respectively, and immunoblot analysis and a standard Dextran Polymer Conjugate Two-step Visualization System Envision was applied in 1 study each. The data of HR and 95% CI was extracted from survival curves or literature reports. In all these studies, 17 studies researched OS [17, 22–37], 7 studies investigated DFS/RFS [22, 23, 26, 38–41] and 6 studies focused on progression-free survival (PFS)/ metastasis-free survival (MFS) [28, 31, 32, 42–44] (Table 2).

**Table. 2:** HRs and 95% CIs of cancer prognosis and progression associated with TSP-1 expression in included studies

First author, Publication year	Cut-off value	Number of cases			OS		DFS/RFS		PFS/MFS	
		High expression	low expression	Total	HR (95% CI)	P Value	HR (95% CI)	P Value	HR (95% CI)	P Value
Nakamura et al., 2019	10% of the cells were positive	86	120	206	N/A	N/A	N/A	N/A	1.11 (0.56–2.19)	0.774
Tzeng et al., 2016	value =40	76	107	183	N/A	N/A	N/A	N/A	0.61 (0.38–0.99)	< 0.001
Rouanne et al., 2016	Median	N/A	N/A	171	0.15 (0.03–0.89)	0.04	0.39 (0.10–1.45)	0.23	N/A	N/A
Teraoku et al., 2016	score = 3	35	59	94	2.61 (1.00–8.16)	< 0.01	0.63 (0.38–1.06)	0.06	N/A	N/A
Campone et al., 2015	positive	19	14	33	0.86 (0.08–8.85)	0.0364	N/A	N/A	N/A	N/A
Eto et al., 2015	10% of the cells were positive	17	48	65	0.53 (0.24–1.18)	< 0.05	N/A	N/A	N/A	N/A
Yao et al., 2014	Median	N/A	N/A	68	1.52 (0.91–3.14)	0.088	1.62 (0.91–3.76)	0.112	N/A	N/A
Sharma et al., 2013	Median	N/A	N/A	67	0.982 (0.541–1.784)	0.953	N/A	N/A	N/A	N/A
Pectasides et al., 2012	Median	60	60	120	1.84 (1.11–3.05)	0.018	N/A	N/A	1.73 (1.11–2.69)	0.016
Nakao et al., 2011	30% of the cells were positive	17	48	65	0.54 (0.26–1.14)	< 0.01	N/A	N/A	N/A	N/A
Zhou et al., 2009	10 percentile	72	8	80	0.41 (0.07–2.38)	0.042	N/A	N/A	N/A	N/A
Randall et al., 2009	score = 3	112	54	166	1.44 (0.70–2.75)	0.32	N/A	N/A	1.30 (0.67–2.54)	0.44
Yang et al., 2009	moderate staining	25	45	70	N/A	N/A	0.77 (0.18–3.42)	0.012	N/A	N/A
Secord et al., 2007	Median	32	35	67	1.93 (1.12–3.32)	0.018	N/A	N/A	2.19 (1.29–3.71)	0.004
Wada et al., 2006	score = 2	9	51	60	N/A	N/A	2.85 (1.05–7.72)	0.689	N/A	N/A
Sutton et al., 2005	Median	45	137	182	1.82 (1.0–3.1)	0.01	N/A	N/A	N/A	N/A
Fontana et al., 2005	positive	54	23	77	N/A	N/A	N/A	N/A	2.25 (0.81–6.27)	0.07
Poon et al., 2004	0.75	15	45	60	2.49 (0.63–9.86)	0.014	N/A	N/A	N/A	N/A
Aishima et al., 2002	50% of the cells were positive	34	33	67	1.39 (0.70–2.78)	0.08	N/A	N/A	N/A	N/A
Maeda et al., 2001	positive	89	61	150	N/A	N/A	2.37 (1.41–3.83)	0.03	N/A	N/A
Straume et al., 2001	moderate staining	77	104	181	2.07 (1.27–3.40)	0.0001	N/A	N/A	N/A	N/A
Kodama et al., 2001	positive	31	23	54	N/A	N/A	3.16 (1.25–7.98)	0.015	N/A	N/A
You et al., 2000	5% of the cells were positive	29	10	39	1.49 (0.48–4.58)	0.0163	N/A	N/A	N/A	N/A
Yao et al., 2000	moderate staining	22	32	54	0.81 (0.14–4.58)	0.045	N/A	N/A	N/A	N/A

OS, overall survival; DFS, disease-free survival; MFS, metastasis-free survival; RFS, recurrence-free survival; PFS, progression-free survival; HR, hazard ratio; CI, confidence interval; N/A, not available

### OS associated with TSP-1 expression

**Fig. 2:**
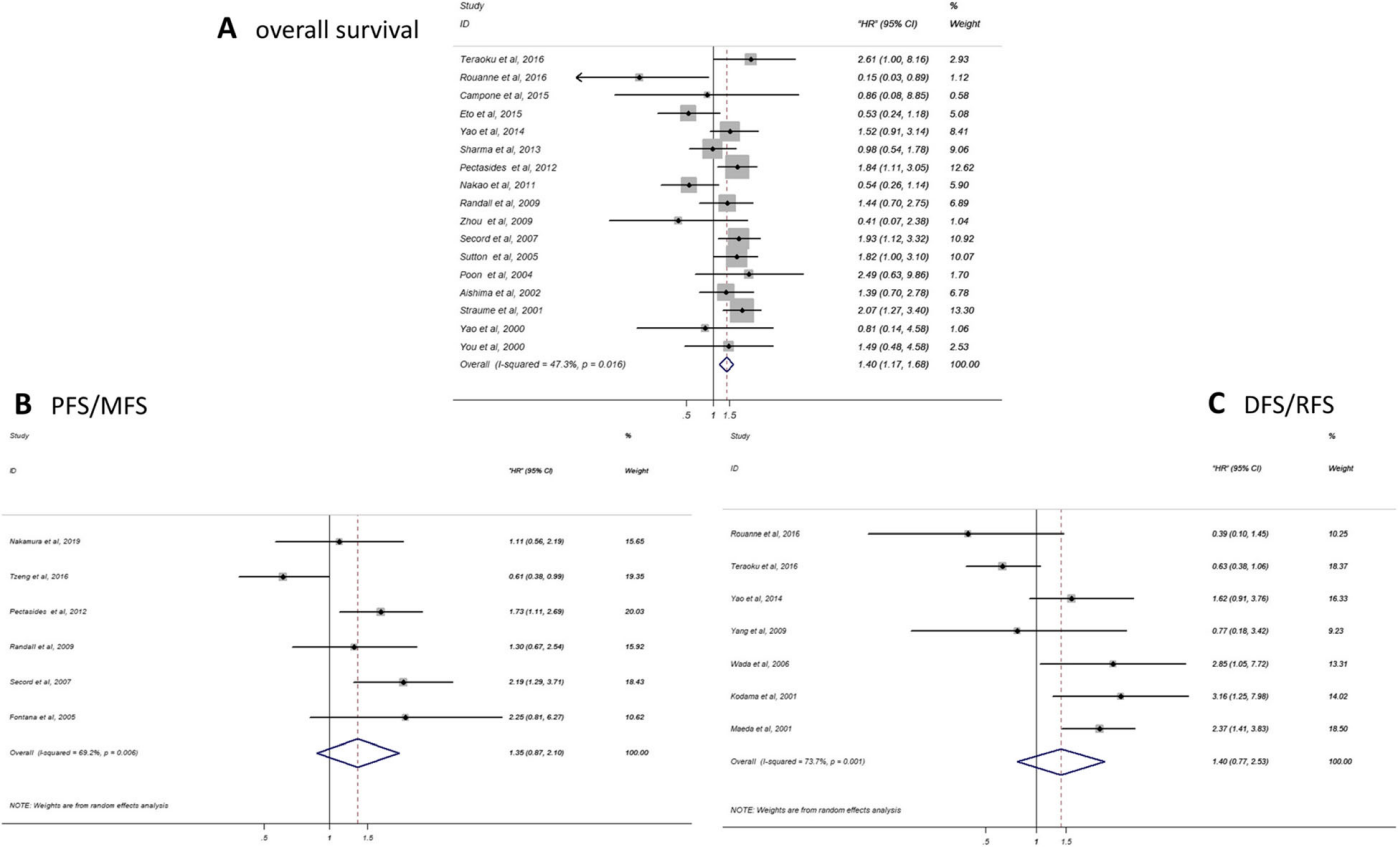
Forest plots of merged analyses estimated for the correlation between survival and TSP-1 expression. (A) Forest plot to assess the OS analysis; (B) Forest plots for the PFS/MFS analysis; (C) Forest plots of the DFS/RFS analysis. Abbreviation: OS, overall survival; PFS, progression-free survival; MFS, metastasis-free survival; RFS, recurrence-free survival; DFS, disease-free survival; HR, Hazard ratio

**Fig. 3:**
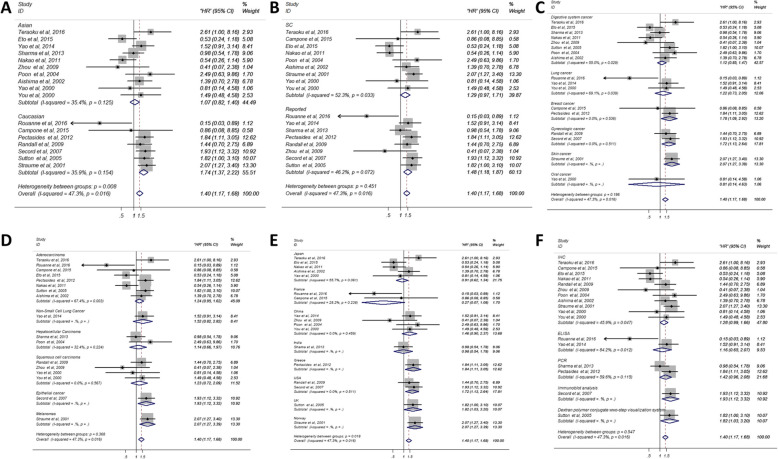
Forest plots of merged analyses evaluated the correlation between OS and TSP-1 expression. (A) Forest plots for the subgroup analysis in different ethnicities; (B) Subgroup analysis in different source of HR; (C) Subgroup analysis in different disease types; (D) Subgroup analysis in different pathological types; (E) Subgroup analysis in different nationalities; (F) Subgroup analysis in different assay methods. Abbreviations: HR, Hazard ratio; SC: survival curve; PCR, polymerase chain reaction; IHC, immunohistochemistry; ELISA, enzyme linked immunosorbent assay

Because of the mild heterogeneity (*p* = 0.016, I^2^ = 47.3), the fixed effect model was used for data analysis. The results showed that high level of TSP-1 indicated poor OS, (HR = 1.40; 95% CI: 1.17 ~ 1.68) and the effect was statistically significant (P<0.001) (Fig. 2A). In order to analyze the source of heterogeneity, we did subgroup analyses according to nationality, dominant ethnicity, main pathological type, disease type, assay method and source of HR. When stratified by ethnicity, we found that the high level of TSP-1 was significantly correlated with the OS of Caucasians (HR = 1.74; 95%CI: 1.37–2.22; P<0.001), while among Asians, there was no significant correlation (HR = 1.07, 95%CI: 0.82–1.40; *P* = 0.629) (Fig. 3A). In the source of HR analysis, the OS of “reported” group was significantly worse (HR = 1.48; 95%CI: 1.18 ~ 1.87; *P* = 0.001), while the OS of the other group was also worse, however, with no statistical significance (HR = 1.29; 95%CI: 0.97 ~ 1.171; *P* = 0.081) (Fig. 3B). According to the subgroup analysis of disease type, the pooled HR of breast cancer was 1.78(95%CI: 1.09 ~ 2.92; *P* = 0.022) (I^2^ = 0.0%, *P* = 0.536), and the pooled HR of gynecological cancer was 1.72(95%CI:1.13–2.64; *P* = 0.012)(I^2^ = 0.0%, *P* = 0.511), with no heterogeneity (Fig. 3C). Finally, there was a significant relationship between elevated TSP-1 and poor OS in Americans. (HR = 1.72; 95%CI: 1.13–2.64; P = 0.012) (Fig. 3E). Other kinds of diseases had no obvious significance (Fig. 3D, F).

### PFS/MFS and DFS/RFS associated with TSP-1 expression

**Fig. 4:**
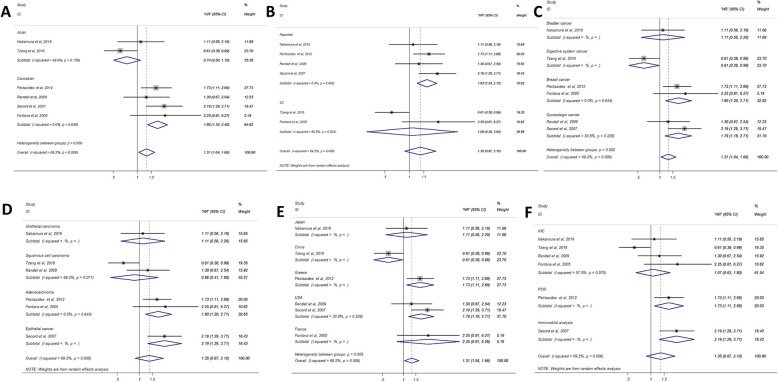
Forest plots of merged analyses for PFS/MFS associated with TSP-1 expression. (A) Forest plots for the subgroup analysis in different ethnicities; (B) Subgroup analysis in different source of HR; (C) Subgroup analysis in different disease types; (D) Subgroup analysis in different pathological types; (E) Subgroup analysis in different nationalities; (F) Subgroup analysis in different assay methods. Abbreviations: HR, Hazard ratio; SC: survival curve; PCR, polymerase chain reaction; IHC, immunohistochemistry

Six studies were included in the PFS/MFS analysis, in which a random-effect model was used because of the significant heterogeneity (*p* = 0.006, I^2^ = 69.2) (Fig. 2B). Our outcomes showed that there was no significant correlation between TSP-1 and PFS/MFS (HR = 1.35; 95%CI: 0.87–2.10; *P* = 0.176). Likewise, subgroup analyses were stratified for the PFS/MFS group to identify the potential source of heterogeneity and other significant information. In ethnicity subgroup, high expression of TSP-1 was related to unfavorable PFS/MFS in Caucasians (HR = 1.80, 95%CI: 1.34–2.40; P<0.001) (Fig. 4A). Stratifying by the source of HR, high TSP-1 expression revealed a significant relationship with poor PFS/MFS, mainly in the report group (HR = 1.63, 95%CI: 1.24–2.15; *P* = 0.001) but not in the SC group (Fig. 4B). The subgroup analysis of cancer type indicated that TSP-1 have a statistically significant association with the breast cancer group (HR = 1.80, 95%CI: 1.20–2.71; *P* = 0.004) and gynecologic cancer group (HR = 1.79, 95%CI: 1.18–2.71; *P* = 0.006) (Fig. 4C). when stratified by main pathological type, analysis in the adenocarcinoma group exhibited a significant correlation between up-regulated expression of TSP-1 and PFS/MFS (HR = 1.80, 95%CI: 1.20–2.71; P = 0.004) (Fig. 4D). Elevated TSP-1 predict poorer PFS/MFS in patients in the USA group (HR = 1.79, 95%CI: 1.18–2.71; P = 0.006) (Fig. 4E). The subgroup analysis in different assay methods had no obvious significance (Fig. 4F).

We analyzed tumor recurrence associated with overexpression of TSP-1 by DFS/RFS. Seven studies focused on DFS/RFS analysis, with a high degree of heterogeneity (*P* = 0.001, I^2^ = 73.7) (Fig. 2C). There was no correlation between high level of TSP-1 and poor DFS/RFS, (HR = 1.40, 95%CI: 0.77–2.53; *P* = 0.271) by random effect model. Furthermore, through the subgroup analyses, we did not observe statistically significant outcomes (Fig. S1). In summary, no relationship was found between DFS/RFS and TSP-1.

### Cumulative meta-analysis

**Fig. 5:**
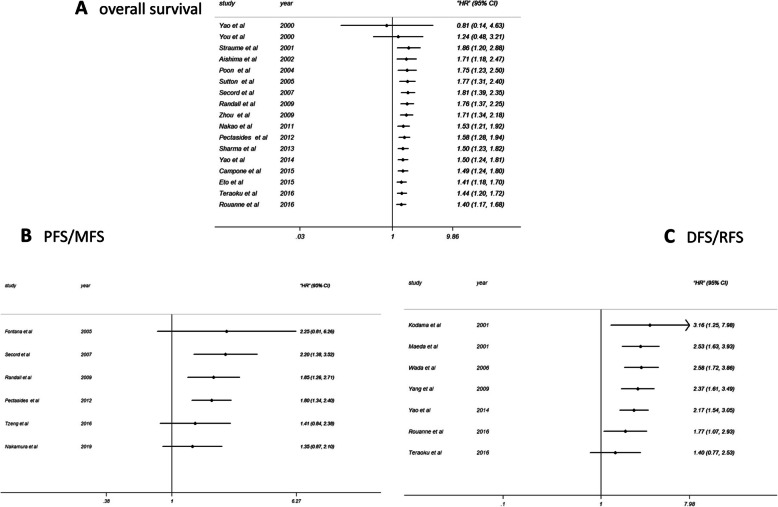
Cumulative meta-analyses for survival associated with TSP-1 expression. (A) Cumulative meta-analysis of OS; (B) Cumulative meta-analysis of PFS/MFS; (C) Cumulative meta-analysis of DFS/RFS. Abbreviation: OS, overall survival; PFS, progression-free survival; MFS, metastasis-free survival; RFS, recurrence-free survival; DFS, disease-free survival; HR, hazard ratio

The main function of cumulative meta-analysis is reflecting the dynamic trend of the research results and evaluating the impact of each research on the comprehensive results. All the selected studies were sorted according to the year of publication. (Fig. 5). The relationship between OS and TSP-1 was first statistically significant in 2001. In addition, the corresponding 95% CIs of OS became narrower with the continuous inclusion of studies, suggesting increasing estimated accuracy. On the contrary, as time goes on, the relationship of TSP-1 and DFS/RFS or PFS/MFS are no longer statistically significant, indicating growing controversy in recent research.

### Publication bias

Egger’s test and Begg’s funnel plot were applied to indicate publication bias in the included studies (Fig. S2). No obvious asymmetry was noticed in funnel plots and the *P* value of Egger’s test also revealed no obvious publication bias. (OS: *P* = 0.066; DFS/RFS: *P* = 0.934; PFS/MFS: *P* = 0.713).

### Sensitivity analysis

**Fig. 6:**
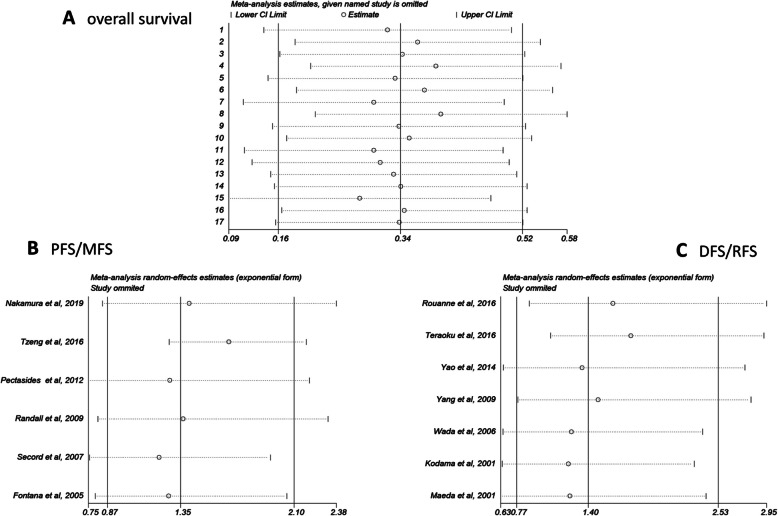
Sensitivity analysis of each included study. (A) Sensitivity analysis of OS for individual studies. (B) Sensitivity analysis of PFS/MFS for individual studies. (C) Sensitivity analysis of DFS/RFS for individual studies. Abbreviations: OS, overall survival; PFS, progression-free survival; MFS, metastasis-free survival; RFS, recurrence-free survival; DFS, disease-free survival

In order to ensure the robustness of the above results and evaluate the stability of results, a sensitivity analysis was carried out by Stata 12.0 software. The analyzed result from a fixed model of OS and two random model of DFS/RFS and PFS/MFS demonstrated that no single study considerably influenced the pooled HRs or 95% CIs, suggesting that the results of the present meta-analysis are credible (Fig. 6).

## Discussion

TSP-1 is a homotrimeric protein which is a member of the thrombospondin gene family [3]. More and more evidence proved that the abnormal expression of TSP-1 is related to the clinical prognosis of cancer patients. Previous research has shown that TSP-1 plays an important role in inhibiting angiogenesis, anti-tumor activity and participating in tissue repair [8]. However, some studies deemed TSP-1 is connected with carcinogenesis [9]. The role of TSP-1 in various cancers has been widely researched, but the conclusions are not consistent.

This is believed to be the first meta-analysis systematically analyzing the association between TSP-1 expression and clinical features of multiple cancers, which included 24 studies with a total of 2379 patients.

Our analysis showed that the pooled HR was 1.40, indicating that the elevated TSP-1 was significantly associated with lower OS (P<0.001). The pooled HR of PFS/MFS analysis and DFS/RFS analysis was 1.35 (*P* = 0.176) and 1.40 (*P* = 0.271) respectively, which demonstrated the consistency of the results, but they were not statistically significant.

Some studies have shown that TSP-1 promotes the development of prostate cancer, and this property is stronger than its anti-angiogenic properties which are mediated by its binding to the CD36 receptor [45, 46]. In addition, they found that TSP-1 promotes cell migration by mediating TRPV3 and, in patients, TSP-1 mRNA level in tumor tissue was significantly associated with PSA relapse. Furthermore, there could be a vicious circle in which TSP-1 inhibits angiogenesis and thus increases hypoxia which induces TSP-1 expression in return to speed up cell migration [10]. All the studies consistent with the results from our analysis. Detection of TSP-1 can identify subgroups of high-risk patients with poor outcomes.

In addition, Kang et al. found Sphingosylphosphorylcholine induces TSP-1 secretion which might play an important role in epithelial-mesenchymal transition since migration and invasion are the key indicators of epithelial-mesenchymal transition [47]. Then, the migration and invasion of breast cancer were significantly interrupted when they knocked down TSP-1. Moreover, in breast cancer patients, the high expression of TSP-1 was significantly associated with poor RFS and MFS [10, 47, 48].

We used subgroup analyses to find out the influence of nationality, dominant ethnicity, main pathological type, disease type, assay method and source of HR on the relationship between TSP-1 and patient prognosis. With regard to the ethnic subgroup analysis, we made a distinction between Asians and Caucasians to clarify the impacts of different genetic backgrounds on the results. Interestingly, the analysis showed a significant association between the high expression of TSP-1 and OS/PFS/MFS (HR = 1.40, 95%CI: 0.77–2.53) (HR = 1.80, 95%CI: 1.34–2.40; P<0.001) in Caucasians, but not in Asians. The reason may be attributed to differences in genetic background and environmental exposure. Secondly, OS and PFS/MFS of breast cancer and gynecological cancer in disease type subgroup were remarkable poor. Campone et al. deem TSP1 as bad prognostic markers by Kaplan-Meier method and immunohistochemistry (IHC) in triple-negative breast cancers [24]. Some studies have shown that TSP-1 promotes the invasiveness of melanoma which declared TSP-1 is a poor prognostic marker [49]. Nevertheless, other research argued that TSP-1 could significantly inhibit the cell viability of Retinoblastoma cells both in vitro and in vivo [50]. To sum up, the prognostic value of TSP-1 may be different in various cancers. However, more studies are required to confirm the clinical significance of TSP-1 in many samples.

Furthermore, the OS (HR = 1.48; 95%CI: 1.18 ~ 1.87; *P* = 0.001) and PFS/MFS (HR = 1.63, 95%CI: 1.24–2.15; P = 0.001) was significantly worse in “reported” group but not in the SC group. Throughout this literature, 13 of the 24 studies with survival information did not provide the HRs, so we could only gather the data from Kaplan-Meier curves. There may be some slight errors between the accurate data and the estimated HRs and their 95% CIs from the Kaplan-Meier curves, leading to unreliable results. In other subgroups, we did not find any statistical significance.

It should be noted that heterogeneity is a potential and critical issue that should not be ignored when discussing the results of Meta-analysis. We believe that the mild heterogeneity observed in OS is acceptable. Moreover, the heterogeneity of PFS/DFS was greatly reduced by subgroup analysis. Sensitivity analysis was performed to assess the reliability of results which revealed that the pooled HR did not significantly change by omitting any individual studies, meaning that the results of this meta-analysis are credible. no evidence of publication bias was noted.

Although this study provided a comprehensive meta-analysis for the prognostic role of TSP-1 in multiple cancers, several limitations do exist. First, Heterogeneity was noted among the selected studies. The existence of heterogeneity may be contributing to the unique characteristics, such as the nationality, dominant ethnicity, the main pathological type, disease type, assay method, source of HR and critical values of TSP-1 expression. Second, it was difficult to determine a standard expression cutoff value because of different cancers, varied assay method and diverse detected sample in the included studies. This could result in bias in the effectiveness of TSP-1 as a prognostic factor. Most studies have established a median expression, IHC intensities or a scoring system as the cutoff value. Therefore, pooled outcomes may be greater or lower than the actual value and cause bias in the results. What’s more, the relatively small number of studies on PFS/MFS and DFS/RFS is a limitation, further studies with more selected data and enrolled patients are needed. As mentioned above, the data in SC group extracted through Kaplan-Meier curves may not be accurate. Finally, only English articles are accepted in this article, which may cause deviations in the results. These limitations should be addressed in further research and be considered when drawing conclusions.

In conclusion, TSP-1 might serve as an effective index in evaluating the progress and prognosis of multiple cancers, especially in breast and gynecologic cancer, and may be utilized to improve targeted therapies. In order to accurately evaluate the role of TSP-1 as a prognostic factor, more clinical studies are required before TSP-1 is applied, especially for single type of cancer.

## Conclusion

In this paper, we firstly evaluated whether TSP-1 was an accurate prognostic prediction for multiple cancers. Our data provided convincing evidence that high expression of TSP-1 was associated with adverse cancer prognosis. However, further in-depth and larger-scale studies are needed to support our results.

## Supplementary information

**Additional file 1 Fig. S1**: Forest plots of merged analyses for DFS/RFS associated with TSP-1 expression. **Fig. S2**: Begg’s funnel plots of the publication bias

## Data Availability

All data generated or analyzed during this study are included in this manuscript.

## Abbreviations

TSP-1: Thrombospondin-1
HRs: Hazard ratio
CIs: Confidence intervals
OS: Overall survival
PFS: Progression-free survival
MFS: Metastasis-free survival
DFS: Disease-free survival
RFS: Recurrence-free survival

## References

[1] Bornstein P, Sage EH (1994). Thrombospondins. Methods Enzymol.

[2] Adams JC (2001). Thrombospondins: multifunctional regulators of cell interactions. Annu Rev Cell Dev Biol.

[3] Adams JC, Lawler J (2011). The thrombospondins. Cold Spring Harb Perspect Biol.

[4] Wang J, Li Y (2019). CD36 tango in cancer: signaling pathways and functions. Theranostics..

[5] Hayat SMG, Bianconi V, Pirro M, Jaafari MR, Hatamipour M, Sahebkar A (2020). CD47: role in the immune system and application to cancer therapy. Cell Oncol (Dordr).

[6] Atanasova VS, Russell RJ, Webster TG, Cao Q, Agarwal P, Lim YZ, Krishnan S, Fuentes I, Guttmann-Gruber C, McGrath JA *et al*. Thrombospondin-1 Is a Major Activator of TGF-β Signaling in Recessive Dystrophic Epidermolysis Bullosa Fibroblasts. J Invest Dermatol. 2019; 139(7):1497–1505.e1495. 10.1016/j.jid.2019.01.011 30684555

[7] Amagasaki K, Sasaki A, Kato G, Maeda S, Nukui H, Naganuma H (2001). Antisense-mediated reduction in thrombospondin-1 expression reduces cell motility in malignant glioma cells. Int J Cancer.

[8] Roberts DD (2008). Thrombospondins: from structure to therapeutics. Cell Mol Life Sci.

[9] Filleur S, Volpert OV, Degeorges A, Voland C, Reiher F, Clezardin P, Bouck N, Cabon F (2001). In vivo mechanisms by which tumors producing thrombospondin 1 bypass its inhibitory effects. Genes Dev.

[10] Firlej V, Mathieu JR, Gilbert C, Lemonnier L, Nakhle J, Gallou-Kabani C, Guarmit B, Morin A, Prevarskaya N, Delongchamps NB (2011). Thrombospondin-1 triggers cell migration and development of advanced prostate tumors. Cancer Res.

[11] Lawler J (2002). Thrombospondin-1 as an endogenous inhibitor of angiogenesis and tumor growth. J Cell Mol Med.

[12] Grutzmacher C, Park S, Zhao Y, Morrison ME, Sheibani N, Sorenson CM (2013). Aberrant production of extracellular matrix proteins and dysfunction in kidney endothelial cells with a short duration of diabetes. Am J Physiol Renal Physiol.

[13] Grossfeld GD, Ginsberg DA, Stein JP, Bochner BH, Esrig D, Groshen S, Dunn M, Nichols PW, Taylor CR, Skinner DG (1997). Thrombospondin-1 expression in bladder cancer: association with p53 alterations, tumor angiogenesis, and tumor progression. J Natl Cancer Inst.

[14] Fontanini G, Boldrini L, Calcinai A, Chine S, Lucchi M, Mussi A, Angeletti CA, Basolo F, Bevilacqua G (1999). Thrombospondins I and II messenger RNA expression in lung carcinoma: relationship with p53 alterations, angiogenic growth factors, and vascular density. Clin Cancer Res.

[15] Streit M, Velasco P, Brown LF, Skobe M, Richard L, Riccardi L, Lawler J, Detmar M (1999). Overexpression of thrombospondin-1 decreases angiogenesis and inhibits the growth of human cutaneous squamous cell carcinomas. Am J Pathol.

[16] Yamashita Y, Kurohiji T, Tuszynski GP, Sakai T, Shirakusa T (1998). Plasma thrombospondin levels in patients with colorectal carcinoma. Cancer..

[17] Straume O, Akslen LA (2001). Expresson of vascular endothelial growth factor, its receptors (FLT-1, KDR) and TSP-1 related to microvessel density and patient outcome in vertical growth phase melanomas. Am J Pathol.

[18] Kasper HU, Ebert M, Malfertheiner P, Roessner A, Kirkpatrick CJ, Wolf HK (2001). Expression of thrombospondin-1 in pancreatic carcinoma: correlation with microvessel density. Virchows Arch.

[19] Tanaka K, Sonoo H, Kurebayashi J, Nomura T, Ohkubo S, Yamamoto Y, Yamamoto S (2002). Inhibition of infiltration and angiogenesis by thrombospondin-1 in papillary thyroid carcinoma. Clin Cancer Res.

[20] Higgins JP, Thompson SG (2002). Quantifying heterogeneity in a meta-analysis. Stat Med.

[21] Egger M, Davey Smith G, Schneider M, Minder C (1997). Bias in meta-analysis detected by a simple, graphical test. Bmj..

[22] Rouanne M, Adam J, Goubar A, Robin A, Ohana C, Louvet E, Cormier J, Mercier O, Dorfmuller P, Fattal S (2016). Osteopontin and thrombospondin-1 play opposite roles in promoting tumor aggressiveness of primary resected non-small cell lung cancer. BMC Cancer.

[23] Teraoku H, Morine Y, Ikemoto T, Saito Y, Yamada S, Yoshikawa M, Takasu C, Higashijima J, Imura S, Shimada M (2016). Role of thrombospondin-1 expression in colorectal liver metastasis and its molecular mechanism. J Hepatobiliary Pancreat Sci.

[24] Campone M, Valo I, Jezequel P, Moreau M, Boissard A, Campion L, Loussouarn D, Verriele V, Coqueret O, Guette C (2015). Prediction of recurrence and survival for triple-negative breast Cancer (TNBC) by a protein signature in tissue samples. Mol Cell Proteomics.

[25] Eto S, Yoshikawa K, Shimada M, Higashijima J, Tokunaga T, Nakao T, Nishi M, Takasu C, Sato H, Kurita N (2015). The relationship of CD133, histone deacetylase 1 and thrombospondin-1 in gastric cancer. Anticancer Res.

[26] Yao L, Dong H, Luo Y, Du J, Hu W (2014). Net platelet angiogenic activity (NPAA) correlates with progression and prognosis of non-small cell lung cancer. PLoS One.

[27] Sharma BK, Srinivasan R, Kapil S, Singla B, Chawla YK, Chakraborti A, Saini N, Duseja A, Das A, Kalra N (2013). Angiogenic and anti-angiogenic factor gene transcript level quantitation by quantitative real time PCR in patients with hepatocellular carcinoma. Mol Biol Rep.

[28] Pectasides D, Papaxoinis G, Kotoula V, Fountzilas H, Korantzis I, Koutras A, Dimopoulos AM, Papakostas P, Aravantinos G, Varthalitis I (2012). Expression of angiogenic markers in the peripheral blood of docetaxel-treated advanced breast cancer patients: a Hellenic cooperative oncology group (HeCOG) study. Oncol Rep.

[29] Nakao T, Kurita N, Komatsu M, Yoshikawa K, Iwata T, Utsunomiya T, Shimada M (2011). Expression of thrombospondin-1 and ski are prognostic factors in advanced gastric cancer. Int J Clin Oncol.

[30] Zhou ZQ, Cao WH, Xie JJ, Lin J, Shen ZY, Zhang QY, Shen JH, Xu LY, Li EM (2009). Expression and prognostic significance of THBS1, Cyr61 and CTGF in esophageal squamous cell carcinoma. BMC Cancer.

[31] Randall LM, Monk BJ, Darcy KM, Tian C, Burger RA, Liao SY, Peters WA, Stock RJ, Fruehauf JP (2009). Markers of angiogenesis in high-risk, early-stage cervical cancer: a gynecologic oncology group study. Gynecol Oncol.

[32] Secord AA, Darcy KM, Hutson A, Lee PS, Havrilesky LJ, Grace LA, Berchuck A (2007). Co-expression of angiogenic markers and associations with prognosis in advanced epithelial ovarian cancer: a gynecologic oncology group study. Gynecol Oncol.

[33] Sutton CD, O'Byrne K, Goddard JC, Marshall LJ, Jones L, Garcea G, Dennison AR, Poston G, Lloyd DM, Berry DP (2005). Expression of thrombospondin-1 in resected colorectal liver metastases predicts poor prognosis. Clin Cancer Res.

[34] Poon RT, Chung KK, Cheung ST, Lau CP, Tong SW, Leung KL, Yu WC, Tuszynski GP, Fan ST (2004). Clinical significance of thrombospondin 1 expression in hepatocellular carcinoma. Clin Cancer Res.

[35] Aishima S, Taguchi K, Sugimachi K, Asayama Y, Nishi H, Shimada M, Sugimachi K, Tsuneyoshi M (2002). The role of thymidine phosphorylase and thrombospondin-1 in angiogenesis and progression of intrahepatic cholangiocarcinoma. Int J Surg Pathol.

[36] You J, Zhang X, Zhang J, Chen H, Liu Y, Sun L (2000). Expression of thrombospondin-1 and CD44 in human lung cancer tissues and their prognostic significance. Zhongguo Fei Ai Za Zhi.

[37] Yao L, Zhao YL, Itoh S, Wada S, Yue L, Furuta I (2000). Thrombospondin-1 expression in oral squamous cell carcinomas: correlations with tumor vascularity, clinicopathological features and survival. Oral Oncol.

[38] Yang S, Guo LJ, Tang XF, Tan K, Gong RG, Li A, Yu T, Gao QH, Xuan M, Wen YM (2010). The alteration of Id-1 and TSP-1 expression in mucoepidermoid carcinoma associated with its clinical features and prognosis. Int J Oral Maxillofac Surg.

[39] Wada H, Nagano H, Yamamoto H, Yang Y, Kondo M, Ota H, Nakamura M, Yoshioka S, Kato H, Damdinsuren B (2006). Expression pattern of angiogenic factors and prognosis after hepatic resection in hepatocellular carcinoma: importance of angiopoietin-2 and hypoxia-induced factor-1 alpha. Liver Int.

[40] Maeda K, Nishiguchi Y, Kang SM, Yashiro M, Onoda N, Sawada T, Ishikawa T, Hirakawa K (2001). Expression of thrombospondin-1 inversely correlated with tumor vascularity and hematogenous metastasis in colon cancer. Oncol Rep.

[41] Kodama J, Hashimoto I, Seki N, Hongo A, Yoshinouchi M, Okuda H, Kudo T (2001). Thrombospondin-1 and -2 messenger RNA expression in invasive cervical cancer: correlation with angiogenesis and prognosis. Clin Cancer Res.

[42] Nakamura Y, Miyata Y, Takehara K, Asai A, Mitsunari K, Araki K, Matsuo T, Ohba K, Sakai H (2019). The pathological significance and prognostic roles of Thrombospondin-1, and −2, and 4N1K-peptide in bladder Cancer. Anticancer Res.

[43] Tzeng HT, Tsai CH, Yen YT, Cheng HC, Chen YC, Pu SW, Wang YS, Shan YS, Tseng YL, Su WC (2017). Dysregulation of Rab37-mediated cross-talk between Cancer cells and endothelial cells via Thrombospondin-1 promotes tumor Neovasculature and metastasis. Clin Cancer Res.

[44] Fontana A, Filleur S, Guglielmi J, Frappart L, Bruno-Bossio G, Boissier S, Cabon F, Clezardin P (2005). Human breast tumors override the antiangiogenic effect of stromal thrombospondin-1 in vivo. Int J Cancer.

[45] Dawson DW, Volpert OV, Pearce SF, Schneider AJ, Silverstein RL, Henkin J, Bouck NP (1999). Three distinct D-amino acid substitutions confer potent antiangiogenic activity on an inactive peptide derived from a thrombospondin-1 type 1 repeat. Mol Pharmacol.

[46] Dawson DW, Pearce SF, Zhong R, Silverstein RL, Frazier WA, Bouck NP (1997). CD36 mediates the in vitro inhibitory effects of thrombospondin-1 on endothelial cells. J Cell Biol.

[47] Kang JH, Kim HJ, Park MK, Lee CH (2017). Sphingosylphosphorylcholine induces Thrombospondin-1 secretion in MCF10A cells via ERK2. Biomol Ther (Seoul).

[48] Sid B, Langlois B, Sartelet H, Bellon G, Dedieu S, Martiny L (2008). Thrombospondin-1 enhances human thyroid carcinoma cell invasion through urokinase activity. Int J Biochem Cell Biol.

[49] Jayachandran A, Anaka M, Prithviraj P, Hudson C, McKeown SJ, Lo PH, Vella LJ, Goding CR, Cebon J, Behren A (2014). Thrombospondin 1 promotes an aggressive phenotype through epithelial-to-mesenchymal transition in human melanoma. Oncotarget..

[50] Chen P, Yu N, Zhang Z, Zhang P, Yang Y, Wu N, Xu L, Zhang J, Ge J, Yu K (2016). Thrombospondin-1 might be a therapeutic target to suppress RB cells by regulating the DNA double-strand breaks repair. Oncotarget..

