# Supplementary figures and images for: Role of TSP-1 as prognostic marker in various cancers: a systematic review and meta-analysis
Source: BMC Med Genet. 2020 Jun 29;21:139. doi: 10.1186/s12881-020-01073-3 (PMC7325168; doi:10.1186/s12881-020-01073-3)

## Slide 1
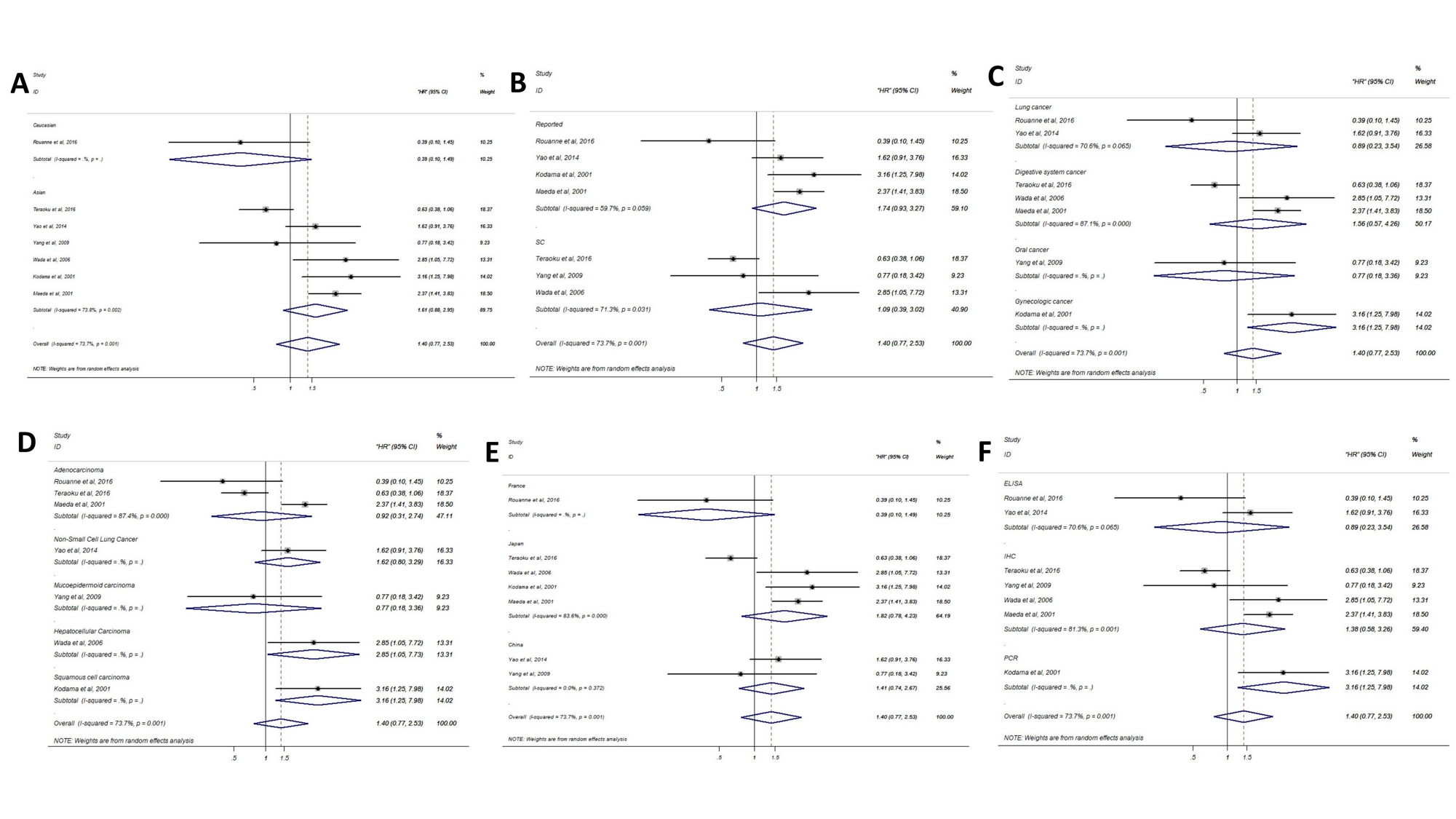

## Slide 2
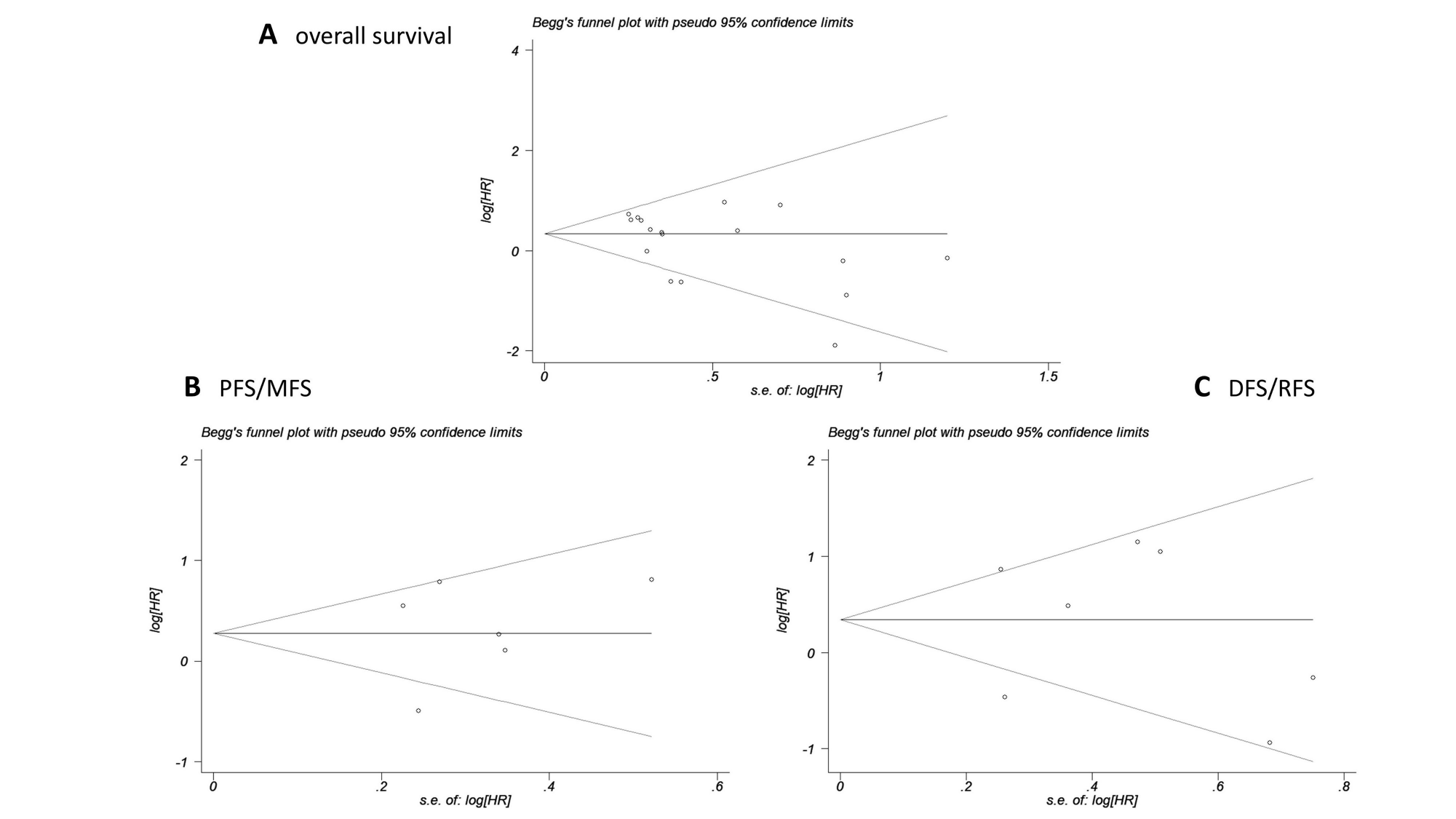

Supplement: Supplementary file 1 — Additional file 1 Fig. S1: Forest plots of merged analyses for DFS/RFS associated with TSP-1 expression. Fig. S2: Begg’s funnel plots of the publication bias [file 12881_2020_1073_MOESM1_ESM.pptx]
